# Exploring the Role of the SLC12A Cation-Coupled Chloride Cotransporters in the Cardiovascular System

**DOI:** 10.3390/cells15171549

**Published:** 2026-08-27

**Authors:** Adriana Mercado, M. Adrián Gutiérrez-Alejo, Sabina Orozco-Navarro, Paola de los Heros

**Affiliations:** 1 Departamento de Bioquímica, Instituto Nacional de Cardiología Ignacio Chávez, Tlalpan, Mexico City 14080, Mexico; sabina.orozco@uabc.edu.mx; 2 Unidad de Investigación UNAM-INC, Facultad de Medicina, Universidad Nacional Autónoma de México, Mexico City 04510, Mexico; gamadlejo@unam.edu

**Keywords:** cation–chloride coupled cotransporters, cardiomyocytes, heart, WNK kinases, ion homeostasis, cardiac health

## Abstract

Cardiomyopathies are a major category of cardiovascular disease, significantly contributing to global morbidity and mortality. Their pathogenesis involves a complex interplay of genetic and environmental factors, with multiple gene families playing critical roles in disease progression. Among these, the Solute Carrier 12A (SLC12A) family encodes membrane cation-coupled chloride cotransporters (CCCs), which are essential for ion transport and cellular homeostasis. By regulating intracellular chloride and potassium concentrations, SLC12A/CCC transporters may directly influence myocardial electrophysiology, repolarization dynamics, and susceptibility to cardiomyopathies. Dysfunction of potassium-handling cotransporters can disrupt action potential propagation, increasing the risk of arrhythmias, while chloride transport abnormalities exacerbate ischemic injury by impairing cell volume regulation and metabolic responses to hypoxia. This review examines the expression and protein levels of SLC12A/CCCs in cardiac tissue, providing crucial insights into their functional significance for maintaining cardiac ion homeostasis. Understanding the regulatory mechanisms of these transporters could inform therapeutic interventions to restore ion balance and mitigate myocardial dysfunction in affected patients, thereby improving cardiac health.

## 1. Introduction

The electroneutral cation-coupled chloride cotransporters (CCCs) belonging to the Solute Carrier 12A (SLC12A) gene family consist of nine transmembrane protein members. Two Na-K-Cl cotransporters—SLC12A1/NKCC2 and SLC12A2/NKCC1—one Na-Cl cotransporter (SLC12A3/NCC), and four K-Cl cotransporters (SLC12A4-7/KCC1-KCC4) are all well characterized. Additionally, there are two orphan members, SLC12A8/CCC8 and SLC12A9/CCC9, for which their specific physiological roles and transported substrates have not yet been identified [1,2]. With the exception of CCC8 and CCC9, CCCs are proteins responsible for the symport of sodium and/or potassium ions coupled with chloride that use the electrochemical gradient created by the Na/K-ATPase to translocate the ions across the membrane without changing its potential, hence their electroneutrality [1,2,3].

CCCs play a crucial role in maintaining cellular ion homeostasis, and their expression patterns have been extensively studied across tissues and organs [1]. One particularly intriguing area of investigation is CCC expression in the heart. As a vital organ responsible for pumping blood throughout the body, the heart relies on the precise control of ion transport mechanisms to maintain proper cardiac function [4,5]. CCCs regulate sodium, potassium, and chloride ions, which are electrolytes essential for cardiac electrical signaling, contractility, and overall cardiac performance [2,6]. Additionally, given their role in cell volume regulation, CCCs may be important in cardiac remodeling. Changes in cell volume are closely linked to proliferation, differentiation, and apoptosis, and cardiomyocyte hypertrophy and cardiac fibroblast proliferation are central to pathological remodeling. Therefore, as key regulators of cell volume, CCCs could influence these remodeling processes by altering intracellular ionic and osmotic homeostasis [7].

The heart, as an electrically active organ, requires precise control of intracellular ion levels, which are vital for proper excitation–contraction coupling, action potential generation, and cellular homeostasis. Because CCCs are key regulators of intracellular chloride concentration, and because this anion affects the shape and duration of cardiac action potentials through various channels and transporters [8], it is reasonable to expect that disruptions in chloride balance may be associated with cardiac arrhythmia and altered contractile function. Therefore, understanding the expression and roles of CCCs in cardiac tissue will be essential for grasping cardiac electrophysiology and disease mechanisms. There is clear evidence that dysregulation of these transporters is implicated in various cardiovascular disorders, including hypertension [2], and more recent evidence is emerging on CCCs in myocardial infarction and heart failure [6]. Evidence includes expression analyses that reveal their presence and distinct expression patterns in cardiovascular tissues. For instance, NKCC2 and NCC are predominantly expressed in the kidney, contributing to sodium reabsorption, which is crucial for maintaining blood pressure and fluid balance [1,3]. Additionally, NKCC1 and KCCs have been identified in cardiac tissue and are suggested to help maintain proper ion balance during cardiac repolarization [6,9,10,11,12,13,14].

Functional studies have demonstrated that CCCs play critical roles in cardiovascular processes, including vascular tone regulation, electrolyte balance, and myocardial contractility [15,16]. Dysregulation of these transporters can disrupt ion gradients, leading to alterations in cardiac electrical activity and contractile function, which may contribute to the development and progression of various cardiovascular diseases, including arrhythmias, heart failure, and hypertrophy [9,12,17]. Moreover, variations in SLC12A genes have been associated with an increased risk of hypertension and other cardiovascular phenotypes in human populations [18,19,20,21]. Therefore, understanding the expression profiles of SLC12A genes and their proteins in the heart is of significant interest, given their potential implications for cardiac physiology and human pathophysiology.

This review aims to provide a comprehensive analysis of the current data on the expression of cation-coupled chloride cotransporters (CCCs) in the cardiovascular system, particularly in the heart, and their potential functional interactions in maintaining cardiac ion homeostasis. These insights will deepen our understanding of the molecular mechanisms underlying cardiac function and help identify potential therapeutic targets to correct ion imbalances in cardiovascular diseases.

## 2. Search Strategy and Selection Criteria

A non-systematic search for this conceptual review was conducted using PubMed, Scopus, Embase, and Google Scholar, prioritizing original articles, reviews, and international guidelines from inception through November 2025. Search terms included combinations of “cation–chloride cotransporters,” “CCC,” “NKCC,” “NCC,” “KCC,” “SLC12A,” “cardiac,” “cardiomyocyte,” “heart,” “cardiovascular system,” and “proteomics/transcriptomics.” The search targeted studies on ionic and volume homeostasis mediated by the SLC12A/CCC family in the cardiovascular system, CCC expression or function in mammalian cardiac tissue or primary cardiac cells, and the use of molecular or functional techniques. Selected articles were relevant to the mechanistic interactions across various experimental domains, including in vitro molecular biochemistry, animal models of cardiovascular disease, human genetic disorders involving CCCs, clinical data on heart disease, and expression data from public databases such as the Cardiac Proteome (cardiacproteomics.com) and the Human Protein Atlas (proteinatlas.org). Access dates for expression data were from 15 November 2024, to 13 February 2025. Only articles published in English were considered. Instead of conducting a comprehensive systematic review of clinical outcomes, the focus was on high-impact studies, landmark genetic findings, and validated mechanistic data that support a cohesive, interdisciplinary understanding.

## 3. The Cation-Coupled Chloride Cotransporter Family

The cation-coupled chloride cotransporters (CCCs) can be divided into two branches. The Na-driven branch consists of members that translocate ions inward and includes two Na-K-Cl cotransporters, the ubiquitously expressed NKCC1 and NKCC2 located at the apical membrane of the thick ascending limb of Henle’s loop in the kidney, and one Na-Cl cotransporter, NCC, which is exclusively located at the apical membrane of the distal convoluted tubule of the nephron. Together, these proteins are known as NKCCs [22,23].

The K-driven branch comprises four members known as KCCs, which are responsible for the outward movement of potassium and chloride ions. Three KCC members (KCC1, KCC3, and KCC4) are widely distributed throughout the human body [13,24,25,26], whereas KCC2 expression is restricted to neurons of the central nervous system (CNS) [27,28].

Members of the CCC family exhibit a similar structural topology, consisting of 12 highly conserved transmembrane domains (TMDs). The TMDs are flanked by intracellular amino (N)-terminal and carboxyl (C)-terminal domains and share about 25% homology in their amino acid sequence. The expression pattern of each member is distinctive, although degrees of overlap are common between CCC proteins [2,29,30]. CCCs are distinguished by the simultaneous movement of cations (Na and/or K) and anions (specifically Cl) to maintain electrically silent transport where chloride is the only anion involved, and their activity is regulated by a signaling pathway that involves phosphorylation and dephosphorylation processes. These mechanisms, in turn, finely modulate the intracellular chloride concentration ([Cl]_i_) and manage changes in cellular volume. Therefore, CCCs perform some of the most important functions within the organism: the maintenance of cell volume, [Cl]_i_ levels, and K efflux [31].

To fulfill their roles, CCCs are tightly regulated by a complex signal transduction mechanism that mediates the reciprocal yet coordinated phospho-regulation of NKCCs and KCCs [1]. This regulation involves a chloride- and volume-sensitive network of serine/threonine kinases and phosphatases [32,33,34,35], including the With No-lysine (K) kinase family (WNK) [36], the STE-20 proline–alanine-rich kinase (SPAK) and Oxidative Stress Response element 1 (OSR1) kinases [37], and phosphatases, such as protein phosphatase 1 (PP1), which counterbalance kinase activity [38,39]. Therefore, because CCCs are widely expressed throughout the organism and are essential for many physiological functions, impairment of any of these proteins or their regulators is critically implicated in many human renal and electrolyte disorders, such as Gitelman’s syndrome, Bartter’s syndrome [18,19,20,21], and Gordon’s syndrome [40,41], as well as neurological diseases, including Andermann’s syndrome [42], schizophrenia, autism, and epilepsy [43], some forms of cancer, and other cardiovascular diseases, including coronary heart disease, stroke, heart failure, peripheral arterial disease, arrhythmia, and congenital heart disease [1,2,3].

Despite their physiological importance, CCCs remain understudied compared with other protein groups. Their role in the cardiovascular system is increasingly recognized, as they are thought to play a critical role in regulating cardiac metabolism and contractile function. As a result, CCCs are emerging as new targets for metabolic interventions to prevent or treat cardiovascular disease.

### 3.1. Functions and Expression of the Cation-Coupled Chloride Cotransporters

Cation-coupled chloride cotransporters are essential for maintaining transepithelial ion absorption and secretion, cellular ion balance, osmotic regulation, and pH homeostasis in various tissues and cell types [1,2,44].

Sodium–(potassium)–chloride cotransporters, or N(K)CCs, mediate the simultaneous and silent transport of cations (Na and/or K) and Cl across the membrane in a Na-dependent manner, thus being part of numerous physiological processes such as volume regulation, cell-shrinkage-induced Na influx, and the maintenance of electrical excitability in neuronal pathways [1,2,44]. N(K)CCs are particularly important in the kidneys, where they contribute to the homeostasis of the intracellular Cl concentration and cell volume and are fundamental in transepithelial ion secretion and the reabsorption of sodium, chloride, and other ions from the glomerular filtrate back into the bloodstream [3]. These processes are critical for maintaining electrolyte balance, blood pressure, and overall fluid homeostasis [3]. Moreover, NCC and NKCC2 are targets of thiazides and loop diuretics, widely used to treat hypertension and edema, respectively [45,46,47]. NKCCs can also influence intracellular pH regulation by transporting chloride ions. Following stimulation, NKCC activity increases intracellular Na concentration and cell volume, which in turn stimulates the Na/K-ATPase, leading to reduced intracellular ATP levels and altered intracellular pH due to the disruption of ion-coupled transport, mainly by the Sodium–Hydrogen Exchanger (NHE) and the Sodium-Bicarbonate Cotransporter (NBC) [48]. In cardiac cells, elevated sodium levels can lead to energetic deficits. For example, in conditions such as heart failure or ischemia, overactivation of NKCC1 leads to sodium and chloride accumulation, inducing cellular edema and initiating a cascade of ionic imbalances, resulting in exacerbated cell damage and metabolic impairment [49,50,51,52].

As part of the CCC family, the potassium-chloride cotransporters (KCCs) mediate the silent, coupled transport of K and Cl across the membrane, independent of Na. Like the N(K)CCs, these cotransporters are implicated in numerous important physiological processes, including volume regulation, cell-swelling-induced K efflux, and the maintenance of electrical excitability in the CNS [12,13,44,53,54]. Overall, KCCs are expressed in various organs, tissues, and cells, including the heart, kidney, epithelia, brain, skeletal muscle, red blood cells, and endothelial vasculature, where they play roles in transepithelial salt absorption, cell volume regulation, and the regulation of extracellular and intracellular K and Cl, as well as renal K secretion [12,55,56].

Except for KCC2, the net K and Cl efflux pathway that translocates chloride out of the cell through KCCs remains inactive under normal physiological conditions [28,57,58]. However, the function of all KCCs is favored by various stimuli, such as increased cell volume, modifications of thiol groups in the presence of N-ethylmaleimide (NEM), and cell acidity [12,13,44,53]. It has also been suggested to result from adrenergic stimulation [12,17,59], thereby maintaining ion balance within cells and tissues that contribute to the regulation of intracellular chloride concentration in exchange for potassium, which is essential for proper cellular functions, including osmotic regulation, cell volume decrease, membrane potential, and inhibitory/excitatory signaling [12,13,44,53].

In the kidney, KCCs are expressed throughout the nephron, where KCC3 has been implicated in glucose reabsorption in the proximal tubule [60,61], in maintaining cell volume during enhanced Na/HCO_3_ transport in the intercalated cells [61,62], and in Cl recycling, which can be coupled to the Cl/HCO_3_ exchanger pendrin in the distal tubule, contributing to K loss [61,63]. In the kidney, KCC4 is expressed in the distal nephron, where it is involved in salt reabsorption in the thick ascending loop of Henle and in acid-base balance in the collecting duct cells, where it plays a role in acid secretion and intracellular pH [48,60,64].

Outside the brain and kidneys, KCCs have also been found in many tissues, including the intestine, liver, and heart, where they are involved in transepithelial salt absorption and in regulating extracellular and intracellular K/Cl [12,56]. KCC4 is expressed abundantly as KCC1 in the muscle, brain, lungs, heart, and kidneys, where it helps maintain acid-base equilibrium (i.e., its absence can lead to cardiac arrhythmias) and recycles chloride that enters the cell via the basolateral Cl efflux pathway through anion exchangers in acid-secreting epithelial cells [12,13,65,66]. Furthermore, a K-Cl cotransport has been proposed in cardiomyocytes, where it may play a role in regulating cardiac activity, affecting heart rate variability and overall cardiovascular health [9,17,67]. It is also thought to serve as a compensatory mechanism that counteracts Cl^−^ overload under β-adrenergic stress-induced pathological conditions, thereby contributing to cell volume regulation [12]. While its precise role in cardiac physiology remains unclear, heart rate regulation is influenced by direct cardiac mechanisms and neurotransmitter systems, underscoring the complexity of cardiovascular control.

Consequently, alterations in the expression and function of cation-coupled chloride cotransporters (CCCs) have been associated with a range of conditions, including neurological and psychiatric disorders, ion flux imbalances, and cardiovascular conditions. This highlights the broad physiological significance of CCCs and the importance of the tightly regulated activity of all members of this protein family.

### 3.2. Regulation of Cation-Coupled Chloride Cotransporters by Phosphorylation

The cation-coupled chloride cotransporters (CCCs) are widely distributed glycoproteins that work together to maintain cellular volume homeostasis and ion transport. To this end, CCC function is finely modulated by a (de)phosphorylation cascade orchestrated by protein kinases and phosphatases, which reciprocally stimulate one branch of the family while inhibiting the other [1,68,69,70,71,72,73,74]. The main regulators of the CCCs are the WNK [with no lysine (K)] serine/threonine kinases, which consist of four isoforms (WNK1-WNK4) [75]. Within the catalytic domain of WNKs, subdomain II contains a unique substitution in which a highly conserved lysine residue (hence their name) is replaced by a cysteine [74,76]. Together with the STE20/SPS1-related proline–alanine-rich protein kinase (SPAK) and the oxidative stress-responsive kinase 1 (OSR1), WNKs play a critical role in maintaining cellular homeostasis [2,68,77].

**Figure 1:**
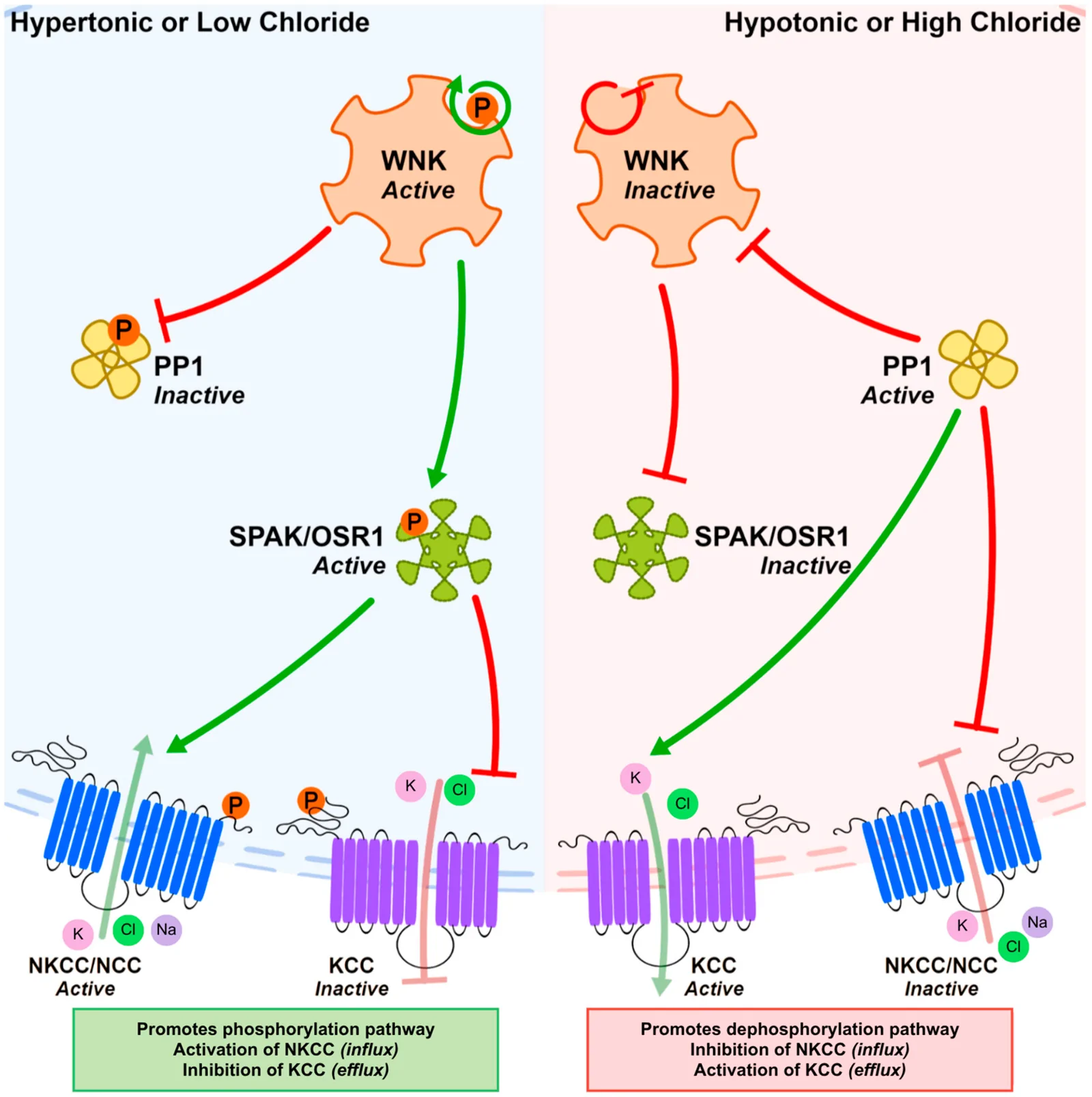
**Phospho-regulation of CCCs via the WNK-SPAK/OSR1-CCC pathway.** In response to hypertonic or low-chloride stimuli (left blue), WNK kinases become activated through autophosphorylation [75], subsequently phosphorylating and activating their main downstream targets, the SPAK/OSR1 kinases [2,68,77]. SPAK/OSR1 phosphorylates the CCCs, thereby activating the transport of Na, Cl, and K ions into the cell via the cotransporters NCC-NKCC1/2. In turn, the K cotransporters (KCCs) are inhibited [70,73,79,81,82]. In response to hypotonic or Cl-increasing stimuli (right red), the WNK-SPAK/OSR1 kinases become dephosphorylated and inactive, marking the onset of PP1 activity [1,68,69], which dephosphorylates the CCCs, promotes extrusion of K and Cl ions via activated KCCs, and inhibits NCC-NKCC1/2 under these conditions.

WNK kinases are activated by autophosphorylation in response to decreased intracellular chloride concentration or osmolarity [78,79,80]. Upon activation, WNKs phosphorylate the downstream SPAK/OSR1 kinases, which in turn phosphorylate CCCs. This phosphorylation activates the Na-driven cotransporters (NKCCs) while reciprocally inhibiting the K-driven (KCCs) members. Conversely, when WNK-SPAK/OSR1 kinases cannot be phosphorylated due to increased intracellular chloride concentrations or hypoosmotic conditions, the upstream CCCs remain dephosphorylated. This state leads to the activation of KCCs and inhibition of NKCCs (Figure 1). This dynamic regulatory mechanism allows CCCs to respond to environmental conditions and cellular demands in a reciprocal manner [70,73,79,81,82]. Therefore, by modulating CCC activity, WNK-SPAK/OSR1 kinases are implicated in various pathologies, including cancer, anemia, neuropathic pain, hypertension, and other cardiovascular disorders [68,69,71,73,82].

Chloride is an important electrolyte involved in cell volume regulation, blood pressure control, and intracellular ion transport, and is also essential for renal and cardiac signaling [83]. Recent studies have shown that hypochloremia, a common condition among patients with acute or chronic heart failure (HF) characterized by low serum chloride levels, is associated with adverse prognosis [84]. It remains unclear how this electrolyte abnormality develops in HF patients and what its causal relationship is with adverse outcomes. Proposed mechanisms include increased activation of the renin–angiotensin–aldosterone system, stimulation of WNK signaling, and adverse effects on myocardial conduction and contractility [85].

As mentioned previously, WNK’s activity is regulated by chloride concentrations. In this way, WNK kinases will sense hypochloremia and will induce increased chloride transport by NCC and NKCC2 in the kidney [79]. Nevertheless, loop diuretics remain a cornerstone of HF management, often requiring high doses to effectively address volume overload and its associated symptoms, such as edema and dyspnea [86,87]. These diuretics exert their effect by directly inhibiting NCC and NKCC2. Activation of these cotransporters by WNK chloride sensing and, at the same time, inhibition by diuretic action will produce a persistent cycle of electrolyte disturbances [86] (Figure 4). As a result, patients with hypochloremia will take higher doses of loop diuretics, yet it is uncertain whether these high doses of diuretics are the cause of hypochloremia or whether their use becomes necessary because of hypochloremia-induced diuretic resistance [88]. Further research into the association between loop diuretics and persistent hypochloremia is needed. There are still many unknowns in the pathophysiology and treatment of hypochloremia and how it affects patients with HF.

A clear example of this modulation was demonstrated by Wilson et al. in 2001 [89], showing that the inherited human monogenic disease familial hyperkalemic hypertension (FHHt), also known as pseudo-hypoaldosteronism type 2 (PHAII) or Gordon syndrome, is caused by mutations in the WNK1 and WNK4 genes that enhance WNK function. This condition is characterized by excessive sodium retention and impaired potassium excretion by the kidneys, sustained by the perpetual activity of the Na-Cl cotransporter (NCC) in the distal convoluted tubule of the nephron [89]. The mutations reported in the WNK1 and WNK4 proteins prevent their degradation, leading to increased sodium retention due to a phosphorylated and constitutively active NCC [90,91]. Consequently, any disturbance in the phosphorylation status of the CCCs can lead to conditions that impact the cardiovascular system and other areas of human health [89].

## 4. The Na-(K)-Cl Cotransporters in the Cardiovascular System

The Na-dependent cotransporters of the CCCs include three membrane proteins responsible for moving Na and Cl, with or without K, across multiple epithelial and non-epithelial cells. This group includes two isoforms of the Na-K-Cl cotransporters (NKCC1 and NKCC2) and one isoform of the Na-Cl cotransporter (NCC), which are responsible for translocating chloride into the cells and inducing the transcellular movement of ions across secretory and absorptive epithelia. Consequently, their involvement in diverse physiological processes may contribute to the pathophysiology of cardiovascular disease.

Overall, NKCCs are well known to contribute to cardiovascular diseases due to their roles in vascular and renal cells, where they regulate potassium or sodium levels. An imbalance either promotes atherosclerosis or is a cause of hypertension, respectively. Multiple lines of evidence indicate that CCCs are particularly significant in pathological cardiac conditions. Using real-time qPCR, Yamamoto et al. reported in 2009 changes in sodium transporter expression in rodents with heart failure and cardiac hypertrophy. Additionally, myocardial dysfunction is associated with significant intracellular sodium accumulation, a key feature of a failing cardiomyocyte that promotes calcium overload. Consequently, upregulation of Na influx transporters during hypertrophy may contribute to remodeling processes, causing contractility issues and promoting arrhythmias [92]. If CCCs contribute to pathological sodium buildup, they could serve as new targets for treating heart failure and ischemic heart disease. Nevertheless, the expression, function, and physiological significance of CCCs in the heart remain poorly understood.

Recent advances in the omics field, particularly proteomics, have opened a broad window through which it is possible to access the numerous proteins present in many, if not all, cells and tissues in the human body. Initiatives such as the Cardiac Proteome (cardiacproteomics.com) [93] and the Human Protein Atlas (proteinatlas.org) [94,95], both large-scale academic projects, aim to map and analyze cardiac protein expression in humans and commonly used model organisms across heart chambers using quantitative proteomics.

Under normal physiological conditions, Litviňuková et al. showed that NKCC1 exhibits strong evidence of both RNA transcripts and protein expression in cardiac tissue, whereas NKCC2 and NCC show negligible expression across all regions of the heart [96] due to their kidney-specific expression.

### 4.1. The Na-K-2Cl Cotransporter NKCC1 in the Heart

NKCC1 (SLC12A2) is expressed in a wide variety of epithelial tissues where it functions as a secretory Na-K-2Cl cotransporter. The NKCC1 isoform was molecularly identified from the shark’s rectal gland in 1994 by Xu et al., and was later found in secretory epithelia localized towards the basolateral membrane. This isoform was detected by Northern blot in all cells and tissues analyzed. It is considered a housekeeping protein implicated in ionic balance and cell volume regulation, and, to date, the most extensively studied CCC isoform in cardiac tissue [23].

**Figure 2:**
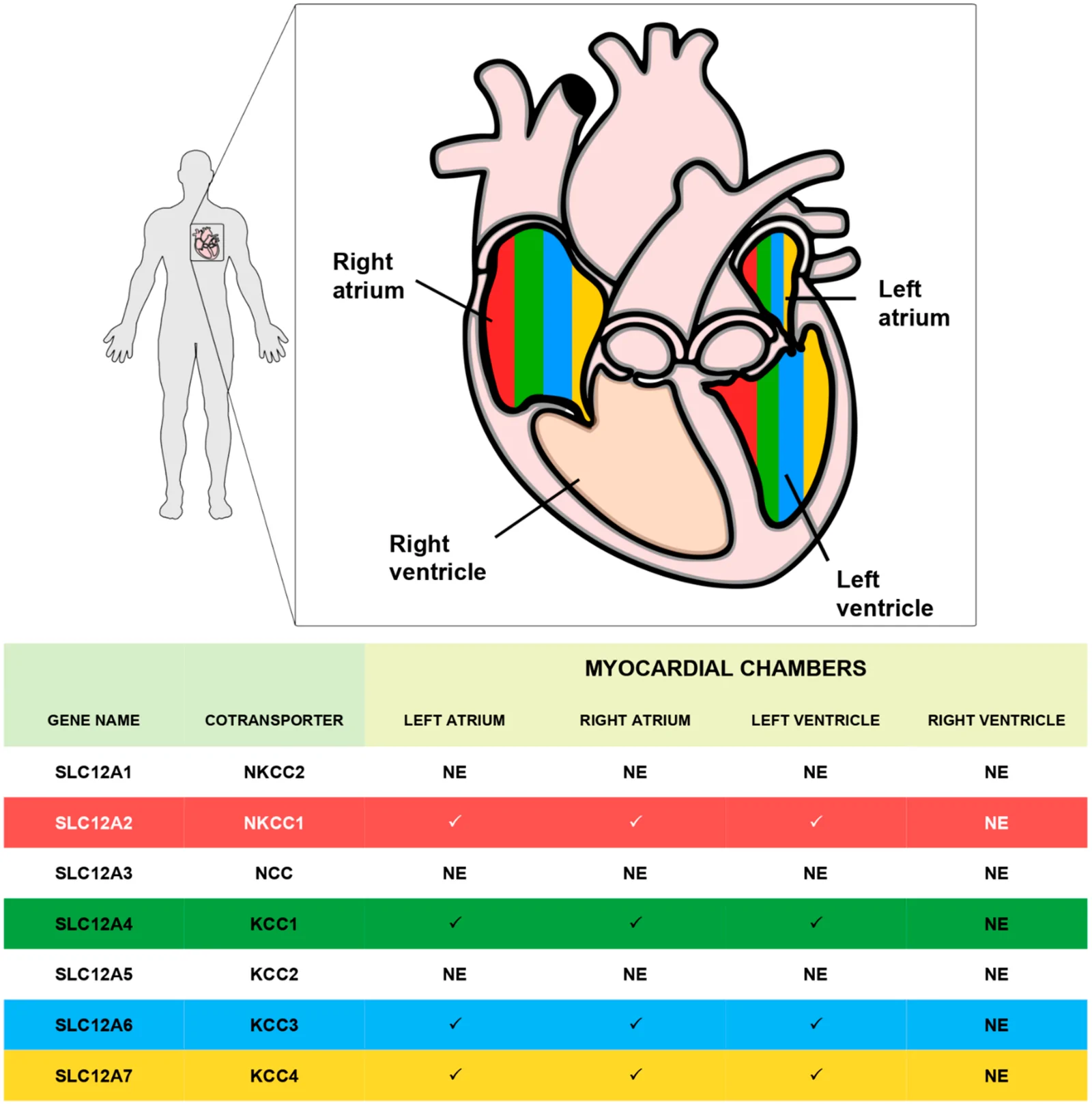
**Cardiac protein expression of human SLC12A/CCCs.** With the exception of NKCC2, NCC, and KCC2, all other cotransporters are expressed within the myocardial chambers: left atrium, left ventricle, and right atrium. None of the CCCs are expressed in the right ventricle. ✔, evidence of expression; **NE**, no evidence of expression. (Red—NKCC1; green—KCC1; blue—KCC3; yellow—KCC4.) Modified from Cardiac Proteome (https://cardiacproteomics.com).

The cardiac proteome shows evidence of NKCC1 in the human heart and in other animal models used for experimentation. According to this database, in human cardiac tissue, the NKCC1 isoform is expressed in the left ventricle, left atrium, and right atrium, while orthologous genes from *Mus musculus*, *Rattus norvegicus*, and *Equus caballus* are also expressed in the right atrium. Indeed, the Human Protein Atlas database also indicates low RNA expression in the heart, particularly in cardiomyocytes (proteinatlas.org) (Figure 2).

Experimental evidence for the functional expression of NKCC1 in the rat heart was initially provided by Andersen et al. between 1998 and 2006 [17,59,97]. Their research demonstrated extensive NKCC1 distribution in normal rat myocardium by immunohistochemistry, with strong expression in cardiomyocytes, and showed that stimulation of α-adrenergic receptors in isolated hearts activates a K efflux pathway that is partially sensitive to loop diuretics and whose phosphorylation and activation are inhibited by calyculin A. This work marked the first indication that rat cardiomyocytes express NKCC1 mRNA transcripts and protein. Furthermore, Prasad et al. in 2008 [10] showed that knockout mice for the Cl/HCO_3_ exchanger (AE3) exhibited increased NKCC1 activity, which involved the influx of Na into myocytes, suggesting that AE3 and NKCC1 are likely to reduce subsarcolemmal [Na]. Moreover, by simultaneously disrupting AE3 and NKCC1, the investigators observed exacerbated myocardial stress following ischemia–reperfusion injury in isolated mouse hearts. The double knockout impaired both myocardial contraction and relaxation *in vivo*. In addition, the study revealed an increase in Ca clearance due to enhanced Na/Ca exchanger (NCX) activity, which directly affected the contractile machinery and overall cardiac performance, emphasizing the critical roles of AE3 and NKCC1 in Ca handling and cardiac function [10].

In hearts from type I diabetic rats, Ramasamy et al. demonstrated a significant increase in cation flux via NKCC1 compared to nondiabetic controls [98]. This enhanced NKCC1 activity alters sodium homeostasis, contributing to ischemic injury in diabetic hearts, and can be mitigated by NKCC inhibitors such as bumetanide. Supporting these findings, biomathematical modeling by Terashima et al. using the Kyoto model—a comprehensive in silico framework that integrates sarcomere shortening and mitochondrial oxidative phosphorylation—has further clarified the role of NKCC1 in maintaining cardiac Cl homeostasis. These findings suggest that NKCC1 functions as a secondary-active cotransporter, contributing to the maintenance of a high intracellular [Cl] of approximately 30 mM alongside passive Cl^−^ currents. Furthermore, the transmembrane movement of alkali metal cations (i.e., Na, K) and Cl ensures optimal cell volume within minutes in cardiac cells [50].

Additionally, in mouse vascular smooth muscle, NKCC1 maintains vascular tone by regulating Cl levels in response to osmotic stress, thereby promoting the contraction of vascular smooth muscle aortic rings (VSMR). However, when NKCC1 activity is inhibited by bumetanide, [Cl]_i_, Ca uptake, and contractions are significantly reduced [6,99]. With the decline in [Cl]_i_, the sarcolemma of VSMR is hyperpolarized, suppressing Ca entry through L-type calcium channels [99,100]. Consequently, VSMR contraction decreases, whereas contraction in cells exposed to sharp depolarization is unaffected by NKCC1 inhibition [6,99].

Therefore, the main roles of the electroneutral Na-K-2Cl cotransporter in the myocardium under physiological conditions are consistent with the regulation of cellular volume to maintain [Cl]_i_ above equilibrium, as previously indicated by Hass in 1994 [51].

Given the physiological and pathological mechanisms of NKCC1 described above, targeting this cation–chloride cotransporter represents a promising therapeutic avenue in cardiovascular disease, although its potential and achievability remain highly context-dependent and hypothetical. Experimental evidence from Andersen et al. demonstrated that bumetanide-mediated inhibition of Na-K-2Cl cotransport during periods of myocardial ischemia and reperfusion impaired Na recovery during reperfusion and was associated with increased intracellular Na and Ca concentrations. These findings suggest that NKCC1 may have distinct functional contributions during ischemia and reperfusion, highlighting an important limitation in translating evidence from renal CCC inhibition to the myocardium: the direction and magnitude of CCC-mediated ion flux may vary with tissue and pathological context. Thus, although clinically available loop diuretics are an established example of CCC modulation, their effects do not constitute evidence for a cardiomyocyte-selective therapeutic strategy. Instead, further characterization of transporter isoform specificity, tissue distribution, pharmacological selectivity, isoform-specific binding sites, and potential systemic, off-target, and electrolyte effects is necessary to translate CCC-directed therapies into cardiovascular interventions [101].

### 4.2. The Na-K-2Cl Cotransporter NKCC2 in the Heart

The NKCC2 isoform (SLC12A1) has been identified only at the protein level in the medullary regions of the thick ascending limb of the loop of Henle in the mammalian nephron [102,103,104]. The anatomic location of NKCC2 expression is critical for regulating extracellular fluid volume and osmolarity. Thus, mutations in NKCC2 in humans are associated with a severe genetic disorder known as Bartter’s syndrome, characterized by hypokalemic alkalosis with hypercalciuria [103].

Evidence of NKCC2 protein expression in human heart tissue is lacking. The cardiac proteome shows no evidence of this cotransporter in human cardiac tissue or in any other animal model used for experimentation (cardiacproteomics.com). However, apical NKCC2 is well-established to play a significant role in NaCl reabsorption in the kidney, contributing to blood pressure maintenance, mitigating the progression of hypertension, and reducing the risk of long-term cardiovascular complications [105].

### 4.3. The Na-Cl Cotransporter (NCC) in the Heart

The Na-Cl cotransporter NCC/SLC12A3 has been identified only within the mammalian nephron, at the apical membrane of the distal convoluted tubule [106]. Although there is some evidence that the NCC in humans and rats is expressed outside the kidney, such as in lens epithelial cells [107], the small intestine (ileum and jejunum) [108], osteoblasts and bone [109], placenta, prostate, colon, and spleen [110], its functional significance awaits deeper investigation.

Gamba et al., using rat tissue and high-stringency assays, failed to detect any NCC mRNA [111]. As with the NKCC2 cotransporter, the cardiac proteome does not show evidence of the Na-Cl cotransporter (NCC) in humans or any other animal model of experimentation (cardiacproteomics.com). Therefore, in mammals, the NCC is expressed exclusively in the kidney, where it regulates NaCl reabsorption and influences blood pressure [22]. Genetic mutations affecting NCC regulation [112] or lower K levels can increase NCC activity, thereby elevating sodium levels and increasing blood pressure [113], which can aggravate cardiovascular problems such as hypertension and heart disease [114].

## 5. The K-Cl Cotransporters in the Cardiovascular System

KCCs facilitate the movement of potassium and chloride ions across epithelial and non-epithelial cells in various organs and tissues. While KCC1, KCC3, and KCC4 are widely expressed [2,3], KCC2 is exclusively localized to the central nervous system [2,3,115,116]. Due to their expression throughout the human body, KCCs regulate ionic homeostasis and cellular volume. Therefore, alterations in their function are the cause of several illnesses, including cardiovascular diseases [6].

**Figure 3:**
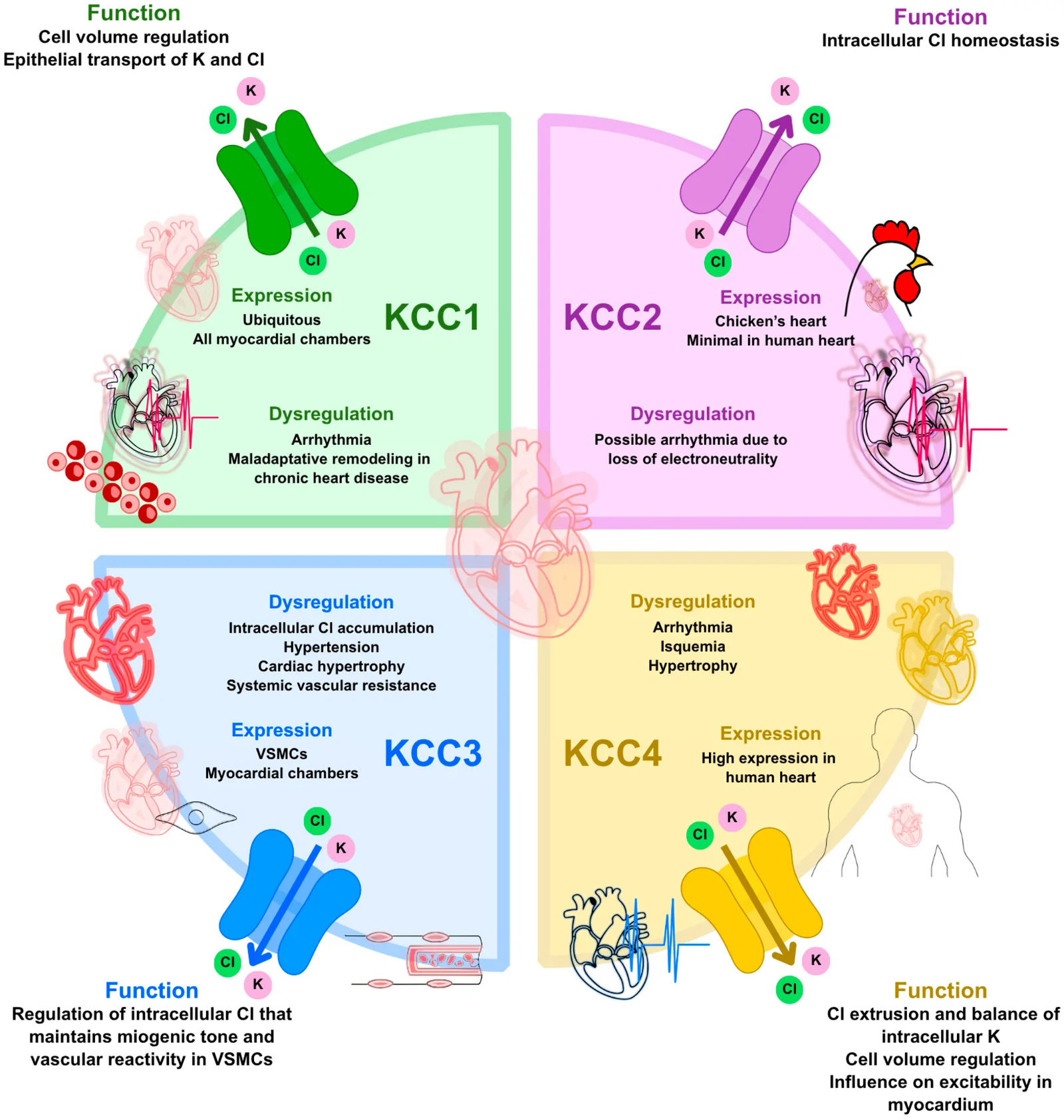
**Expression and function of KCCs in the heart.** KCCs expressed in the myocardium perform functions such as epithelial transport of electrolytes, regulation of intracellular chloride balance, and regulation of cell volume, which are important for maintaining cardiac excitability and myogenic tone. Dysfunction of KCC isoforms contributes to the development of various pathologies, such as arrhythmias, hypertrophy, hypertension, and heart failure [6,9,12,14,17,96,117,118,119,120,121].

Little is known about how KCCs contribute to cardiovascular diseases. They play a significant role in restoring cell volume after a hypoosmotic insult and in regulating potassium and chloride levels, whose imbalance may promote arrhythmogenesis by preventing the cell membrane from being electroneutral. However, the expression and physiological significance of KCCs in the heart are still unclear (Figure 3) [6].

The existence of an electroneutral K-Cl cotransporter in the mammalian heart was first suggested by Yan et al. in 1996 [9]. Their work demonstrated that the extracellular accumulation of K during ischemia in the rabbit myocardium depended, in part, on the contraction of the extracellular space and could not be attributed exclusively to ion channels, suggesting a mechanism of K efflux coupled to Cl without generating a net current. Additionally, the cell-swelling-promoted K efflux pathway they identified was sensitive to bumetanide and NEM, two well-known features of KCCs. Therefore, electroneutral K-Cl cotransporters may play an important role in the development of arrhythmias secondary to cardiac ischemia [9].

### 5.1. The K-Cl Cotransporter KCC1 in the Heart

KCC1 was the first isoform of the KCCs to be molecularly identified in silico by searching the human expressed sequence tag database (ESTdb), revealing a distant relationship to the Na-dependent cotransporters [24]. Analyses of multiple human tissues revealed a ubiquitous expression of KCC1, a housekeeping protein involved in cell volume regulation and ionic homeostasis.

The human heart and cardiac proteome databases show evidence of a wide distribution of KCC1 in the right and left atria, as well as in the left ventricle (proteinatlas.org; cardiacproteomics.com). In addition, using single-cell and single-nucleus transcriptomic analyses of six regions of the human heart, Litviňuková et al. reported that KCC1 is ubiquitously expressed in this organ (Figure 2) [96].

Although KCC1’s role in cardiac tissue remains unclear, it may help maintain the ionic equilibrium of potassium and chloride in cardiomyocytes, which is essential for normal cardiac function. Dysregulation of KCC1 can contribute to altered electrical activity and increased susceptibility to arrhythmias, worsening cardiovascular illness. In addition, excessive expression of the cotransporter could have detrimental effects, such as promoting maladaptive remodeling in chronic heart disease.

### 5.2. The K-Cl Cotransporter KCC2 in the Heart

KCC2 is a unique isoform of the KCCs, exclusively expressed in neurons of the mammalian central nervous system. Two well-characterized isoforms, generated by alternative splicing of the first exon, KCC2a and KCC2b, are distinguished by 40 unique amino acids that KCC2a has at its amino-terminal domain, while KCC2b is shorter. Both isoforms are present in neurons; however, KCC2b has a more significant impact on the organism since it is responsible for intracellular chloride regulation, which is critical for neuronal maturity [115,116].

For many years, KCC2 was assumed to be restricted to neurons. However, a 2012 study by Antrobus et al. showed that KCC2a, the longer splice isoform, is expressed in the chicken heart, particularly in ventricular cardiomyocytes, whereas KCC2b predominates in the chicken brain [12].

Although the role of KCC2a in chicken cardiomyocytes remains unclear, it may be essential for maintaining intracellular chloride homeostasis. As suggested by the authors, KCC2a in chicken cardiomyocytes may provide an important Cl extrusion pathway that counteracts channel-mediated Cl influx (e.g., cystic fibrosis transmembrane conductance regulator [CFTR] and Ca-activated Cl channels) induced by β-adrenergic stimulation. By doing so, this mechanism may help prevent arrhythmogenesis, which occurs when the cell membrane loses its electroneutrality [12,117].

Recently, Litviňuková et al. reported evidence of minimal KCC2 expression in the human heart, present only in cardiomyocytes [96]. In this report, the authors do not specify the isoform present in these cells; therefore, its identity remains unknown. In contrast, the human heart and cardiac proteome databases show no evidence of KCC2 expression in any chamber of the human heart, nor of orthologs in any other species (proteinatlas.org; cardiacproteomics.com). Nevertheless, the cotransporter’s role in ion homeostasis in lower species could indicate a regulatory function in cardiac tissues, similar to its effects on neurons. Physiological processes governed by KCC2, such as chloride transport regulation, are also crucial in cardiomyocytes, suggesting a possible role in cardiac function that has not yet been explored.

To explain the presence of KCC2 in avian cardiac tissue, as demonstrated by Antrobus et al., and its absence in the mammalian myocardium, it is important to consider evolutionary and functional perspectives. In 2015, Hartmann et al. provided a phylogenetic analysis of the KCC family, indicating that in early vertebrates, gene duplication events led to the emergence of distinct isoforms with specialized functions and regulatory properties. Phylogenetic analysis confirms that the four known vertebrate KCC members are derived from a common ancestor that diverged early from the branch containing sodium:potassium:chloride cotransporters (NKCCs). Phylogenetic and syntenic analyses indicate that the four KCC genes arose through two rounds of gene duplication in early vertebrates, followed by lineage-specific losses and retentions. The closest paralogous genes are KCC2 and KCC4, which originated from duplication of the same ancestral gene [29]. Therefore, in the chicken lineage, KCC2 expression expanded to the heart while retaining its ancestral expression pattern, whereas in mammals it became restricted to neurons. Additionally, KCC4, along with other KCCs, may have assumed the cardiac role [12]. This pattern exemplifies sub-functionalization or divergence in gene expression after duplication.

The predominant focus on KCC2 in the nervous system raises questions about its potential functions in other tissues, including the heart. This warrants further investigation into its expression and role in non-neuronal contexts in organisms other than mammals.

### 5.3. The K-Cl Cotransporter KCC3 in the Heart

The third K-Cl cotransporter isoform, KCC3, has several isoforms generated by alternative first exons [118]. Of these, the most studied are KCC3a and KCC3b, which differ in the length of exon 1 at the amino terminus [13,25,26]. Both isoforms are widely expressed, including in the mammalian kidney, brain, and heart, where their specific localization and function remain to be elucidated. Recently, Litviňuková et al. showed that KCC3 (without determining the isoform) is highly expressed in human ventricular cardiomyocytes compared to atrial cardiomyocytes [96]. The cardiac proteome database indicates that KCC3 is expressed in the right and left atrium and in the left ventricle of the human heart, as well as in its ortholog in *Mus musculus* (proteinatlas.org; cardiacproteomics.com) (Figure 2). Mutations in the SLC12A6 gene, which encodes KCC3, are detrimental to human health, promoting a severe sensorimotor neuropathy associated with an intellectual disability known as Andermann syndrome [42]. Mice with disrupted or absent KCC3 exhibit neurological manifestations and hypertension due to thickened vessel walls, which increase blood flow [14,119]. KCC3 is expressed in vascular smooth muscle cells (VSMCs) of various blood vessels, where it regulates the intracellular chloride concentration ([Cl]_i_). Rust et al. demonstrated that the absence of KCC3 in mice significantly elevated [Cl]_i_ in VSMCs without altering K and Na concentrations [119]. Moreover, the chronic arterial hypertension observed in these mice was confirmed by the hypertrophy of resistance vessels, suggesting that KCC3 influences myogenic tone and vascular reactivity by regulating intracellular chloride levels [14,119,120,121].

Garneau et al. demonstrated the role of KCC3 in maintaining vascular homeostasis. Using KCC3 knockout mice, they observed higher diastolic blood pressure and lower pulse pressure than in wild-type mice, as well as increased systemic vascular resistance and left ventricular mass. Their findings suggest that KCC3 contributes to the physiological properties of blood vessels and that, since the cotransporter is expressed in the mouse heart, its absence leads to cardiac hypertrophy, negative chronotropism, and, ultimately, cardiac growth [14].

It is well documented that KCC3 regulates myogenic tone in resistance vessels, and that its absence leads to Cl accumulation in vascular smooth muscle cells, decreasing inward Cl currents and impairing cardiovascular function and homeostasis [14,119]. However, the specific molecular mechanisms through which KCC3 influences these processes remain poorly defined.

### 5.4. The K-Cl Cotransporter KCC4 in the Heart

The last KCC identified at the molecular level was KCC4 (SLC12A7). This isoform is the most sensitive to changes in cell volume and is highly active under hypotonic conditions [13,122,123]. In addition, KCC4 is widely distributed throughout the mammalian body, with the highest expression in the heart, followed by the kidney [13]. According to the Cardiac Proteome database, KCC4 is expressed in the left ventricle and in the right and left atria, and its ortholog is present in *Mus musculus*, *Danio rerio*, and *Rattus norvegicus* (proteinatlas.org). Likewise, the study by Litviňuková et al. indicates that KCC4 is highly expressed in human ventricular cardiomyocytes compared with atrial cardiomyocytes [96], where it may play a significant role in maintaining cardiac function by regulating cell volume, facilitating chloride ion extrusion, and balancing potassium levels, thereby influencing the excitability and contractility of cardiac myocytes (Figure 2).

In humans, KCC4 expression is most abundant in the heart [13]. It has been suggested that the presence of a K-Cl cotransporter may be critical for maintaining Cl homeostasis in cardiomyocytes, helping to counteract the increase in chloride load mediated by Cl channels (such as CFTR and Ca-activated Cl channels) that occurs when the heart rate increases, particularly following β-adrenergic stimulation [12]. Studies using cultured rat myocytes subjected to β-adrenergic stimulation with isoprenaline showed important changes with deleterious effects on the myocardium. These changes include cardiomyocyte apoptosis, disruption in signal transduction, and hypertrophic processes [124], favoring a reduction in both the number and sensitivity of cardiac β-adrenergic receptors, along with an increase in the relative weight of the heart. Additionally, stimulation of α-adrenergic receptors in isolated rat hearts [17] and cell swelling induced by cardiac ischemia activate a K outflow pathway that is partially sensitive to loop diuretics. This pathway may be mediated predominantly by Na-K-Cl and/or K-Cl cotransport mechanisms. According to Yan et al., this K outflow could play an important role in the genesis of arrhythmias followed by cardiac ischemia in a rabbit model [9], suggesting that a KCC cotransporter could be the relevant protein involved.

In summary, the specific role of KCCs in cardiac tissue remains under investigation; however, they are postulated to contribute to regulating intracellular ion concentrations, thereby influencing cardiomyocyte function. The activity of KCCs in extruding K and Cl from cells is essential for cell volume regulation, particularly under conditions of osmotic stress. This mechanism is crucial in cardiomyocytes, where precise control of cell volume and ionic composition is necessary for optimal contractile function. Furthermore, KCCs’ role in maintaining ionic balance may have implications in cardiac electrophysiology. Alterations in intracellular Cl levels can affect the membrane potential and, consequently, the excitability of cardiomyocytes. By modulating Cl extrusion, KCCs may influence cardiac action potential and rhythmicity.

The dysregulation of cation–chloride cotransporters, including KCCs, has been associated with cardiovascular diseases [6,16,45,54]. Although direct studies on KCCs in cardiac tissue are limited, their broader functions in cardiovascular physiology suggest that they are important for maintaining cellular homeostasis. Therefore, further research is needed to explore the specific contributions of various KCC isoforms and their potential as therapeutic targets in cardiovascular diseases.

In mammals, KCC2 has evolved into the neuronal cotransporter responsible for maintaining a low intracellular Cl gradient, which is crucial for GABA- and glycine-mediated synaptic inhibition [28,125,126]. Its promoter and regulation are adapted to rapid neuronal function, and its presence in the adult mammalian heart would be harmful by altering the Cl equilibrium potential and destabilizing the cardiac action potential, given that the Km for Cl, reported in *Xenopus* oocytes by Song et al. as 6.8 mM, is close to the intracellular Cl activity in mature neurons, corresponding to a neuronal efflux mechanism [28]. For this reason, evolution has silenced cardiac KCC2 expression in mammals, although the expression and function of an electroneutral K and Cl cotransporter remain necessary to maintain ionic and volume homeostasis in cardiomyocytes.

Although KCC4 was described as a cell-swelling-activated cotransporter [13,122] and is widely expressed in the mammalian heart [13], it is also modulated by intracellular signaling pathways that would allow it to perform the function that KCC2a fulfills in avian cardiac tissue [12]. β-adrenergic stimulation of monkey COS-7 and human HEK293T cell lines, and mice, activates protein kinase A (PKA), which modulates the WNK/SPAK-OSR1 kinases [127,128]. This signaling pathway, in turn, regulates KCCs, as Antrobus proposed for KCC2a in chicken cardiomyocytes [12]. Consequently, KCC4 could functionally replace KCC2 in the heart, mediating electroneutral K and Cl transport in response to β-adrenergic stress and maintaining ionic homeostasis in the mammalian myocardium.

Unlike KCC2, which has a significantly higher affinity for chloride, KCC4 has a lower affinity for both ions (~17 mM) [122]. This is because KCC4 is physiologically specialized to respond robustly to cell swelling and maintain chloride efflux in specific environments, such as the alpha-intercalated cells of the kidney [60,64] or the inner ear of rats and mice [64].

In mammals, the cellular landscape of the myocardium differs markedly from that of the nervous system. While the neuronal cotransporter KCC2 is absent in mammalian cardiomyocytes, KCC4 is highly expressed in the myocardium, along with KCC1 and KCC3 [24,122]. However, the low-affinity kinetic characteristics of KCC4 are well suited and physiologically optimal for mitigating the consequences of adrenergic stimulation, specifically acting as a protective mechanism against the resulting ischemia and hyperkalemia in this tissue [16].

## 6. Ion and Volume Dysregulation in Cardiovascular Diseases Associated with CCCs

Some cardiac conditions are associated with pathological imbalances in volume and ions, particularly sodium, potassium, and chloride, within cardiomyocytes, which may be closely linked to the function of the cation–chloride cotransporter family. These transporters play a crucial role in maintaining ion homeostasis, which is essential for the proper electrophysiological function of the heart. Disruptions in ion homeostasis can lead to various cardiac pathologies, including arrhythmias, myocardial hypertrophy, ischemic events, and heart failure, among others. The CCC family, particularly the KCC1, KCC3, and KCC4 cotransporters, and NKCC1, is integral to these processes (Table 1) [16,129].

### 6.1. Sodium Imbalance

Pathological sodium (Na) imbalance in the heart is primarily mediated by the Na-K-2Cl cotransporter (NKCC1/SLC12A2), the only member of the SLC12A family that transports Na into cardiomyocytes [17,51]. Normally, intracellular Na remains low (10–15 mEq/L), but during ischemia, heart failure, or hypertrophy in humans and rats, NKCC1 activity and expression rise abnormally (~20 mEq/L) in response to neurohumoral signals such as angiotensin II or catecholamines, as well as to kinases such as PKC and ERK [17,51,59], and the WNK-SPAK kinases [82].

Excessive Na^+^ influx through NKCC1 dangerously elevates the intracellular Na^+^ concentration. In in vivo and in vitro models, this overload forces the Na/Ca exchanger (NCX) to operate in reverse mode, expelling Na and simultaneously absorbing large amounts of calcium [10,130]. The resulting excess of cytosolic and mitochondrial Ca induces cellular damage, activates proteases such as calpain, and triggers cell death in rat ischemia–reperfusion injury [131]. Blocking NKCC1 with bumetanide mitigates changes in intracellular Na levels, thereby protecting the heart against ischemic damage in rat and rabbit hearts [98,101].

In addition, high Na levels and the resulting Ca overload promote sarcoplasmic reticulum leakage and delayed depolarizations, which lead to ventricular arrhythmias, especially in cases of heart failure in rats, rabbits, and humans [92,108,132]. Therefore, NKCC1 inhibition could potentially reduce arrhythmia risk by modulating intracellular ion concentrations and, indirectly, calcium handling.

During ventricular hypertrophy, upregulation of Na influx transporters such as NKCC1 is essential and contributes to cellular remodeling. Yamamoto et al. demonstrated that losartan, an angiotensin II AT1 receptor antagonist, boosts rat NKCC1 levels, thereby modulating pathological growth and fibrosis in the myocardium [92]. Furthermore, blocking the AT_1_ receptor prevents both molecular changes in Na transporters and the onset of pathological hypertrophy, establishing NKCC1 as a potential downstream effector of angiotensin II in cardiac remodeling. Finally, the accumulation of Na also induces cellular edema and contributes to electrical instability within the heart, as demonstrated in isolated cardiac myocytes from chick embryos, rabbits, and guinea pigs [52].

Overall, the overactivation of NKCC1 constitutes a critical mechanism of cardiac injury, and its pharmacological inhibition represents a promising approach to cardio-protection (Table 1).

### 6.2. Potassium Imbalance

The pathological increase in extracellular potassium concentration is a common feature of diverse cardiovascular diseases and is a determinant of the excitability and electrical conductivity of myocardial affections. Experimental and clinical evidence has shown that in conditions such as ischemia, cardiac insufficiency, and hypertrophic cardiomyopathy, ATP production diminishes, compromising Na/K-ATPase activity, facilitating extracellular potassium efflux and cell membrane depolarization, and increasing susceptibility to potentially lethal ventricular arrhythmias [133]. In this respect, although the evidence is limited and more research is still necessary, several members of the CCC family have been suggested to be involved in compensatory mechanisms that aim to restore ionic homeostasis by their coordinated Na, K and Cl cotransport.

In human physiology, the cotransporter KCC4 has been reported to play an important role in acute myocardial ischemia due to its high affinity for potassium. In this cardiovascular disease, rapid extracellular K accumulation results from interrupted coronary flow. This accumulation favors heterogeneous electrical conduction and facilitates the emergence of reentry circuits that underlie ventricular arrhythmias. The activation of KCC4 contributes to ion redistribution, counterbalancing the effects of local hyperkalemia to reestablish electrical stability and reduce the risk of arrhythmia during the acute ischemic event [16].

Heart failure is another cardiovascular disease in which the dysfunction of multiple membrane transporters and ion channels contributes to extracellular potassium accumulation and to alterations in the chloride electrochemical gradient. Myocardial structural and electrical remodeling is accompanied by major alterations in intracellular Na, K, and Cl handling. KCC2 and KCC4 have been shown to play a compensatory role in humans with this condition, favoring coordinated K and Cl extrusion, supporting intracellular chloride homeostasis, mitigating electrophysiological alterations, reducing arrhythmias associated with HF, and preserving contractile function [16].

Moreover, changes in KCC3 isoform protein expression and structural remodeling have been identified in hypertrophic cardiomyopathy in mice. In this pathological condition, KCC3 participates in maintaining cell volume through the regulation of regulatory volume decrease (RVD) and indirectly regulates potassium and chloride balance during hypoxic or ischemic episodes associated with hypertrophic cardiomyopathy. Likewise, KCC3 participates in vascular resistance regulation and hemodynamic overload protection [14].

Finally, cardiac arrhythmias constitute one of the main cardiovascular affections due to intracellular K and Cl homeostatic alterations. Cardiomyocyte intracellular chloride concentration directly influences its electrochemical potential, the activity of voltage-activated chloride channels, and neurohumoral signals. In this respect, Antrobus et al. showed that chloride transport by KCC2a in chicken hearts after β-adrenergic stimulation plays a protective role in cardiac muscle cells by limiting intracellular Cl^−^ concentrations and stabilizing the membrane potential, suggesting that this isoform is important for the electrical responses in the myocardium [12]. Although KCC2 expression in mammalian cardiac tissue remains a matter of debate, these findings support the hypothesis that K-Cl transport can regulate the susceptibility to develop cardiac arrhythmia in certain cardiovascular diseases (Table 1).

In conclusion, the evidence presented here suggests that KCC2, KCC3, and KCC4 participate in the homeostatic mechanisms responsible for sustaining cardiomyocyte ionic equilibrium when facing a pathological increase in extracellular K. Despite their different functions and transport characteristics, they all contribute to the maintenance of membrane potential, regulation of cell volume, and electrical stability of the myocardium.

### 6.3. Chloride Imbalance

Chloride homeostasis is a fundamental component of cardiac biophysics; however, it is frequently overlooked. To maintain [Cl]_i_ above the electrochemical equilibrium, it has been predicted that, in cardiomyocytes, the coordinated action of influx and efflux electroneutral transporters is required: the Na-K-2Cl cotransporter (NKCC1) serves as the primary chloride loader, while the K-Cl cotransporters (KCC1, KCC3, KCC4) function as extruders [134,135]. Therefore, proper action by the CCCs would maintain cellular homeostasis and avoid the consequences of chloride dysregulation, which can lead to detrimental events.

The pathophysiological consequences of hyperchloremia and hypochloremia significantly affect cardiac tissue stability. In humans, serum chloride levels above 106 mEq/L indicate *hyperchloremia*, often associated with dehydration, whereas levels below 96 mEq/L indicate *hypochloremia*. Because CCCs are present in the myocardium, they may play a role in maintaining the cardiac balance of this anion [16,129]. Therefore, disruption of chloride transport can alter action potentials, contributing to arrhythmogenic events [129], and elevated intracellular chloride concentrations can impair cardiac contractility and electrical stability, exacerbating heart failure symptoms [8].

Andersen et al. demonstrate that, in rats, NKCC1 exhibits increased expression in cardiomyocytes after myocardial infarction, suggesting a direct link between elevated intracellular chloride levels and heart failure mechanisms [97]. Furthermore, studies have shown that intracellular chloride concentration rises during ischemia, contributing to arrhythmias.

During myocardial ischemia, oxygen supply is reduced, and oxidative phosphorylation halts [136]. With decreased ATP levels, the Na/K-ATPase is inactivated, [Na]_i_ accumulates, and the Na/Ca exchangers (NCX) reverse, promoting the influx of water and solutes [130]; concomitantly, NKCC1 becomes hyperactive, allowing ions to enter the cell. As intracellular sodium accumulates, chloride ion levels rise, increasing cellular volume. This may activate KCCs, thereby reducing internal osmotic pressure to prevent cell lysis due to swelling, counteracting extreme membrane depolarization, and reducing the occurrence of lethal ventricular re-entry arrhythmias [16,137].

In heart failure, there is chronic hyperactivation of catecholamines and the renin–angiotensin–aldosterone system (RAAS) [138,139]. Continuous β-adrenergic receptor stimulation activates Cl channels, resulting in increased basal Cl influx into cardiomyocytes. To prevent chloride accumulation from altering the ion equilibrium potential, the stabilizing action of electroneutral potassium-chloride cotransporters has been proposed. Therefore, in the heart, the presence of a KCC could constitute an important pathway for Cl efflux from cardiomyocytes, counteracting the channel-mediated accumulation of Cl when the heart rate increases in response to β-adrenergic stimulation [12].

Additionally, KCCs may prevent chronic cellular volume overload in muscle fibers, thereby preventing the accelerated progression toward pathological ventricular remodeling, hypertrophic cardiomyopathy, and loss of contractile force [16].

Although the pathological increase in intracellular chloride concentration in cardiomyocytes is often associated with detrimental outcomes, such as cardiac conditions like arrhythmias and heart failure, some studies suggest that transient elevations may contribute to signaling pathways that could be protective under certain conditions, such as Ischemic Preconditioning (IPC) in rabbit hearts [140], severe metabolic acidosis in rats, mice and rabbits [141,142], and the early phase of myocardial ischemia–reperfusion (I/R) injury in rats [143]. This duality highlights the complexity of chloride’s role in cardiac health and disease [129,144] (Table 1).

**Table 1:** **Activity associated with CCCs during cardiovascular ion imbalance**. During ion imbalance, the activity of cation–chloride cotransporters (CCCs) shifts drastically to restore normal electrolyte levels, intracellular chloride concentration and cell volume.

Ion	CCC/Gene	Expression h = Human m = Mouse r = Rat	Normal Function Associated with the CCC in the Heart	Impact of Ion Imbalance in Cardiovascular Disease	CCC Mechanism Associated with Ion Imbalance	References
**Sodium (Na)** Std. [Na]_i_ = 10–15 mEq	NKCC1/ SLC12A2	Heart (h)(m)(r) VSMC (m)	Influx Na, K and Cl to maintain ion homeostasis	- Heart failure - Ischemia	- Increase in NKCC1 activity promotes Na influx - Accumulation of [Na]_i_ - Reverse activity of NCX and accumulation of [Cl]_i_ - Modifies polarization of the cell	[10,99,130,132,133,135]
**Potassium (K)** Std. [K]_i_ = 150 mEq	KCC1/ SLC12A4	Heart (h)	Reduces internal osmotic pressure by cell swelling	- Ischemia - Cardiac insufficiency - Ventricular arrhythmia	- Coordinates K and Cl efflux, regulating cell volume (KCC) - Restores membrane potential (KCC) - Ion’s redistribution during hyperkalemia and ischemia, restoring electrical potential (KCC4) - Regulation of the RVD during hypoxic and ischemic events (KCC3)	[9,14,16,119,120,121,136]
	KCC3/ SLC12A6	Heart (h) VSMC (m)				
	KCC4/ SLC12A7	Heart (h)(m)(r)				
**Chloride (Cl)** Std. [Cl]_i_ = 10–30 mEq	NKCC1/ SLC12A2	Heart (h)(m)(r) VSMC (m)	Equilibrium of cations and Cl inward and outward of the cell Regulation of the activity in CCCs	- Ischemia - Myocardial infarction - Heart failure - Impaired contractility and ventricular remodeling - Hypochloremia - Hyperchloremia	- The activation of NKCC1 increases cell swelling by influx of Na and Cl, and promotes osmotic stress - KCC counterbalances accumulation of Cl due to efflux of K and Cl - Regulation of ion homeostasis in the heart (NKCC1, KCCs)	[12,16,98,99,129,137,138,139,141,142,143,144]
	KCC1/ SLC12A4	Heart (h)				
	KCC3/ SLC12A6	Heart (h) VSMC (m)				
	KCC4/ SLC12A7	Heart (h)(m)(r)				

### 6.4. Volume Dysregulation

It is important to mention that CCCs have been implicated in atherogenesis, a process that may extend beyond their canonical ion-transport functions and directly intersect with key cardiovascular pathways. Recent work by Pyrpyris et al. on Lectin-like oxidized low-density lipoprotein receptor-1 (LOX-1), the main endothelial receptor for oxidized low-density lipoprotein (LDL), has established that its activation triggers Nicotinamide Adenine Dinucleotide Phosphate (NADPH) oxidase-dependent oxidative stress, endothelial Nitric Oxide Synthase (eNOS) uncoupling, and Nuclear Factor kappa-light-chain-enhancer of activated B cell (NF-κB)-driven inflammation, creating a self-perpetuating cycle of endothelial dysfunction [145]. Under these conditions, LOX-1-derived reactive oxygen species have the potential to alter the phosphorylation status of volume-sensitive CCCs, such as the Na-K-2Cl cotransporter NKCC1, leading to pathological Na^+^ and Cl overload and endothelial cell swelling. These ionic and volume disturbances worsen oxidative stress and increase the expression of adhesion molecules and pro-inflammatory cytokines, creating a feedback loop between LOX-1 signaling and CCC dysregulation.

Beyond the endothelium, CCCs contribute to vascular smooth muscle cell (VSMC) responses to atherogenic stimuli. In 2003, Zhang et al. demonstrated that platelet-derived growth factor (PDGF-BB), a critical mediator released within the plaque microenvironment, activates K-Cl cotransport in VSMCs through a mechanism dependent on reactive oxygen species and tyrosine kinase activity. This activation facilitates K and Cl efflux, resulting in a regulatory volume decrease. Such volume regulation facilitates the migration and proliferation of VSMCs, essential processes for neointima formation and plaque progression. Other growth factors associated with atherosclerosis, including IGF-1, also stimulate K-Cl cotransport in VSMCs. Furthermore, nitrosative species such as peroxynitrite modulate KCC activity within the vascular wall, thereby reinforcing the link between CCCs and the oxidative and inflammatory cascades that promote vessel wall remodeling [146].

Furthermore, genetic models have established a direct role for K-Cl cotransport in maintaining cardiovascular homeostasis. Rust et al. showed that disruption of the K-Cl cotransporter KCC3 in mice results in arterial hypertension via neurogenic mechanisms, accompanied by increased vasoconstriction and vascular remodeling. This finding associates a specific CCC isoform with blood pressure dysregulation, a major risk factor for atherosclerosis, and indicates that KCC3 affects the cardiovascular system through both local vascular ion handling and systemic hemodynamic stress [119].

Overall, these findings suggest that CCCs serve as central hubs for integrating oxidative, growth-factor, inflammatory, and hemodynamic signals within the vessel wall. The LOX-1/NKCC1 signaling loop, PDGF- and IGF-1-dependent activation of K-Cl cotransport in VSMCs, and the hypertensive phenotype resulting from KCC3 disruption highlight the crucial roles of CCC-mediated ionic and volume regulation in endothelial dysfunction and vascular remodeling. A deeper understanding of these ionic mechanisms could provide valuable insights into the complex interplay among metabolic, mechanical, and inflammatory factors that contribute to atherogenesis.

## 7. The WNK-SPAK/OSR1 Signaling Pathway in the Cardiovascular System

WNK expression is widespread throughout the human body. WNK1 is considered a housekeeping protein, with abundant expression in the testis, kidney, and heart, with moderate levels in cardiomyocytes. WNK2 is abundant in the brain [142] and heart [147] but shows low expression levels in cardiomyocytes, whereas WNK3 and WNK4 are widely expressed in the human body, but neither has been detected in cardiomyocytes (proteinatlas.org).

WNK kinases are key regulators of ion homeostasis, sensing chloride and potassium concentrations in body fluids. By directly influencing the tone of blood vessels and blood pressure, they extend their actions not only to the kidney but to the cardiovascular system. Their relevance was highlighted by the generation of global as well as endothelial-specific Wnk1-null mice, which die in utero from embryonic angiogenesis, and cardiovascular and cardiac development defects. These defects include pericardial edema and hemorrhage [148,149,150]. Notably, the Osr1-ablated mice exhibit a phenotype like that of Wnk1-deletion, which was rescued by expressing a constitutively active Osr1 transgene [149].

WNK1 is primarily involved in blood pressure regulation through the WNK-SPAK/OSR1 signaling pathway, which stimulates the activity of the renal Na-Cl cotransporter [151]. This mechanism is significant in the context of hypertension, in which mutations in human WNK1 can lead to pseudo-hypoaldosteronism type II (PHAII) [152].

In relation to the cardiovascular system, several studies have shown the important role of the WNK-SPAK/OSR1-CCC signaling pathway. The long WNK1 isoform (L-WNK1) and WNK3 have been shown to be expressed in the mammalian heart and blood vessels, in both endothelial and vascular smooth muscle cells (VSMCs), during development and adulthood [153]. Its role in the maintenance of vascular tone was first described in the work by Bergaya et al. where it was shown that in a WNK1 +/− mouse model, vascular function is regulated by L-WNK1 via a vasoconstriction pathway that involves α-adrenergic receptors and L-WNK1 effectors SPAK and NKCC1 [154]. Resistant arteries of WNK1 +/− mice showed a significant reduction in their contractile response after phenylephrine treatment. The direct consequence of this reduction was a significantly decreased hypertensive stress response. WNK1 +/− mice exhibited normal basal blood pressure and α1-adrenergic receptor expression. Nevertheless, reduced SPAK phosphorylation affects TRPC6 channel activation, altering sodium and calcium transport into the cell, which is necessary for membrane depolarization and contraction. This study showed an essential role for WNK1 in the vasoconstriction response and regulation of blood pressure after sympathetic nervous system stimulation. In 2012, Susa et al. also showed decreased NKCC1 phosphorylation in the aorta and reduced myogenic tone in the mesenteric arteries of WNK1 +/− mice [153]. Their results indicated that in smooth vascular muscle, NKCC1 activation is dependent on WNK1 expression and regulation. In addition, NKCC1 phosphorylation and responses to phenylephrine and bumetanide were reduced in SPAK knockout (KO) mice, demonstrating that, in vascular smooth muscle cells, the WNK-SPAK-NKCC1 pathway is a determinant of vascular tone regulation [153].

Finally, WNK1 kinase was also shown to display a vasoactive chloride sensor function in endothelial cells (ECs) [155]. As mentioned previously, WNK1 is expressed in these blood vessel cells that regulate arterial contraction. Since chloride anion concentrations are high in ECs and WNK kinases are regulated by their binding, the authors analyzed the role of WNK1 signaling in the arterial contractility of KO mice. In the presence of the vasodilator acetylcholine, the EC Ca-activated Cl channel, TMEM16A (transmembrane protein 16A), becomes activated, reducing intracellular chloride concentrations—a signal that, as mentioned before, activates WNK1, increasing the phosphorylation of its substrate protein OSR1, which in turn increases transient receptor potential vanilloid 4 (TRPVM4) activity, raising intracellular Ca concentrations and ultimately inducing artery relaxation. This response was abolished when WNK1 was inhibited or when intracellular chloride was chelated. In summary, this study identified the role of the WNK1 kinase chloride sensor in the vascular endothelium, where WNK1 senses chloride signals and transduces them, promoting vasodilation and thereby regulating vascular tone [155].

The WNK3 isoform has also been shown to regulate vascular tone through the WNK3-SPAK-NKCC1 cascade. By activating NKCC1-mediated intracellular chloride transport, WNK3 helps maintain high chloride levels in vascular smooth muscle cells, necessary for blood vessel contraction following membrane depolarization [156]. Uchida et al. studied this pathway in mouse aortic tissue and its regulation by dietary salt intake, discovering a new mechanism by which angiotensin II regulates vascular tone [157]. In their study, they found that in high-salt-fed mice, phosphorylation of SPAK and NKCC1 in the aorta was reduced, whereas in mice fed a low-salt diet, it was increased, demonstrating that aortic WNK-SPAK-NKCC1 signaling is regulated by dietary salt intake. The significance of this pathway was demonstrated by studies showing that WNK3 KO mice fed a low-salt diet and treated with angiotensin II showed no increase in SPAK and NKCC1 phosphorylation, indicating that WNK3 signaling is necessary for the response to dietary salt intake in the mouse aorta. WNK3 KO mice also displayed reduced vascular tone and were unable to upregulate blood pressure in response to angiotensin II infusion [158]. Subsequently, Uchida et al. demonstrated in cultured mouse vascular smooth muscle (MOVAS) cells that WNK3-mediated regulation of vascular tone in response to AngII is mediated by Kelch-like protein-2, an E3 ubiquitin ligase adapter protein expressed in the mouse aorta and MOVAS cells [157].

**Figure 4:**
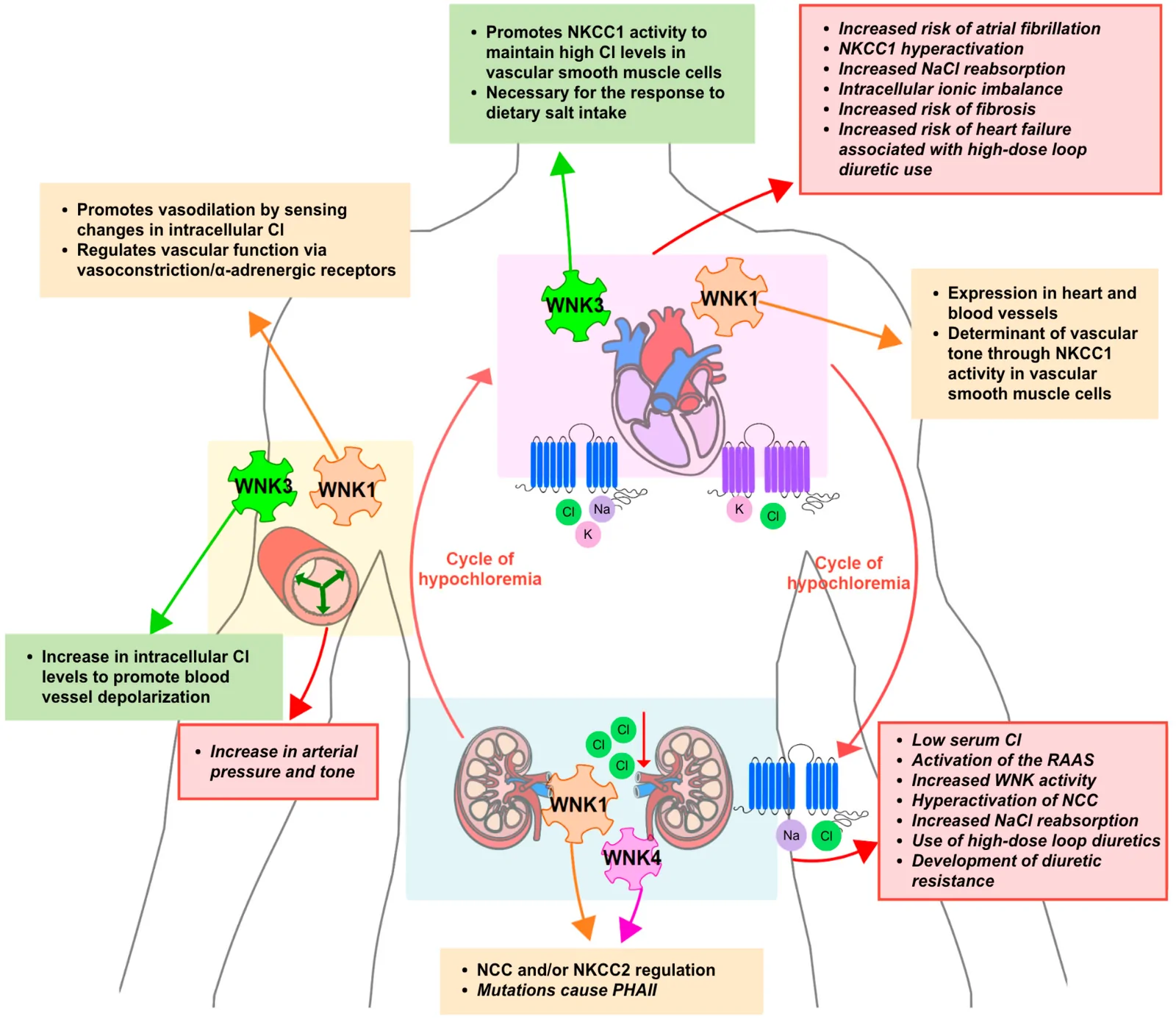
**The role of WNK kinases in the cardiovascular system and in hypochloremia.** In the kidney, WNK kinase activity (primarily WNK1 and WNK4) modulates the activation of Na cotransporters (NCC, NKCC2—blue bars) by sensing a decrease in intracellular Cl [2,68,77]. In the kidney, mutations in WNK are associated with the development of type II pseudo-hypoaldosteronism (PHAII), also known as Gordon syndrome, which causes elevated blood pressure [151,152]. In the cardiovascular system, WNK1 and WNK3 regulate the activity of NKCC1 and the KCCs, primarily by regulating intracellular Cl levels through a balance between the influx activity of NKCC1 and the efflux activity of the KCCs [153,154,155,156,157,161], both of which are important for maintaining vascular tone and vasoconstriction. In the hypochloremia cycle (red arrows and tables), the drop in surrounding Cl triggers activation of WNK kinases in the kidney, which in turn activate the NCC cotransporter and increase Na uptake. The increase in NCC activity is associated with a rise in blood pressure [79,86,88].

On the other hand, recent studies have shown that the WNK-SPAK-CCC signaling pathway is regulated by high dietary K intake. High K intake is inversely related to the occurrence of cardiovascular diseases and mortality and is recommended for the treatment and prevention of hypertension [159]. Likewise, this ion is recognized as a vasodilator due to its effects on vascular smooth muscle and peripheral vascular resistance [160]. Since potassium’s health benefits were reported, the mechanism underlying its antihypertensive effects has been investigated. Given that WNK3 is expressed in vascular smooth muscle cells and that the signaling cascade is activated in response to extracellular potassium, Uchida et al. analyzed the effect of high potassium concentrations on the WNK-SPAK-NKCC1 signaling pathway in the MOVAS cell line to provide a possible mechanism for the antihypertensive effect observed in a high-potassium diet [161]. In this study, the authors reported that when MOVAS cells were exposed to a high-potassium medium, WNK3 protein levels and the phosphorylation of WNK3-SPAK-NKCC1 were reduced. This reduction resulted from increased KLHL2 protein levels in the presence of high potassium concentrations. KLHL2, as an adaptor for the Cullin-3 E3 ubiquitin ligase complex, regulates WNK3 degradation, suggesting that WNK3 plays an important role in the response to high K concentrations and vascular tone in MOVAS cells. KLHL2 knockdown experiments restored WNK3 protein expression and WNK3-SPAK-NKCC1 phosphorylation levels, confirming that the high-potassium medium effect is attributable to KLHL2 activity [161]. In conclusion, this study helps to explain how K might regulate peripheral resistance by providing a mechanism for vascular smooth muscle cells in which a high extracellular K concentration activates NKCC1 phosphorylation through a process involving KLHL2 and WNK3, and demonstrates the WNK-SPAK-NKCC1 signaling pathway’s important role in vasoconstriction regulation (Figure 4).

The previously reviewed series of studies presents a broader and more complete picture of how WNK kinases regulate vascular function through the cation–chloride cotransporters, primarily NKCC1. It seems that vascular cells use a specific WNK isoform to respond to different stimuli; i.e., WNK1 responds to nervous system signals through α1-adrenergic receptors, couples Ca concentrations to vascular contraction via SPAK-TRPV6 signaling, and also acts as a chloride sensor in the endothelium, transducing this ion depletion as a vasodilation response. Moreover, the WNK3 isoform responds to extracellular K concentrations, directly depolarizing the smooth vascular cell membrane via its intermediary, KLHL2. These differential responses are highly significant and could aid the development of more specific and selective drugs for treating vascular diseases. In conclusion, research on the role of WNK kinases in the cardiovascular system is proving that these proteins are not merely renal “salt sensors” but specialized ones that allow blood vessels to integrate and respond to their chemical, hormonal, and electrical environment.

In light of the physiological and pathological roles of the cardiovascular system, regulation of the WNK-SPAK/OSR1 pathway emerges as a promising hypothesis for indirect CCC therapeutic intervention, although it remains largely hypothetical. Inhibition of this signaling cascade, particularly through experimental compounds such as STOCK1S-50699 [162], which targets the conserved carboxy-terminal (CCT) domain of SPAK, could provide a means of modulating multiple CCC-dependent pathways [163]. In principle, reducing NKCC1-mediated Na and Cl influx while simultaneously enhancing K and Cl efflux through KCCs could help restore ionic homeostasis under pathological conditions. In ischemic or hypertrophic myocardium, such modulation may contribute to limiting intracellular Na and Ca accumulation, cellular edema, and electrical instability, thereby potentially preserving cardiac contractility and reducing arrhythmogenic and cell-injury mechanisms. However, WNK-SPAK/OSR1 is ubiquitously present; therefore, achieving sufficient pharmacological selectivity remains a major consideration. Further characterization of isoform-specific signaling, tissue distribution, pharmacological selectivity, and binding sites will therefore be necessary to determine whether this pathway can be safely exploited as a cardiovascular therapeutic target [10,16,70,130].

## 8. Future Research and Conclusions

The study of cation-coupled chloride cotransporters in the cardiovascular system is crucial for understanding physiological and pathological processes, comprehending disease mechanisms, and developing potential therapeutic strategies.

In this respect, several promising directions are being covered for future investigation. First, we can mention dissecting the role of NKCC1 in vascular tone and hypertension. While the kidney’s role in blood pressure regulation via NCCs and NKCC2 is well established, the work presented here highlights the importance of NKCC1 in vascular smooth muscle cells. Several responses in NKCC1 activity and even epigenetic regulation have been observed under high- and low-blood-pressure animal models and in NKCC1-null mice [6], proving the role of NKCC1 in the contribution to vascular tone; nevertheless, future research on NKCC1 in the cardiovascular system is needed to clarify its role in vascular cells, the maintenance of myogenic tone, and the regulation of blood pressure.

Second, analysis of the recently described cryo-EM structures of all CCCs has supplied important and specific information about CCC regulatory domains, composition, conformations, ion-binding sites, and selectivity, providing an excellent opportunity for the development of specific drugs that can target a particular CCC, such as NKCC1 during chronic heart failure or hypochloremia, without targeting NKCC2 [30]. Given CCC’s wide protein expression and functions in the human body, it is important to design highly selective drugs directed to a specific cotransporter to reduce unspecific targeting and secondary effects. This remains an active field of study, with significant areas for improvement in the design of cryo-EM structure-based drugs and therapies for heart failure and cardiovascular diseases.

Third, modulating post-transcriptional mechanisms and upstream regulatory pathways that control CCC activity may provide an indirect, context-specific therapeutic strategy. For instance, SIRT7-mediated regulation of KCC4 stability and activity has been demonstrated experimentally, suggesting a potential modulation cascade for Cl homeostasis without directly targeting CCCs [164]. Similarly, the WNK-SPAK/OSR1 pathway represents a promising target given its ability to regulate multiple SLC12 cotransporters. Experimental inhibition of SPAK, including compounds such as STOCK1S-50699 [162], could theoretically reduce NKCC1-mediated cation influx and favor KCC-mediated K/Cl efflux, thereby restoring ionic homeostasis during cardiac stress through two complementary mechanisms [70]. However, the widespread expression and systemic functions of these regulatory pathways introduce additional challenges for pharmacological selectivity [30,164,165].

Fourth, a meta-analysis of heart rate regulation showed that SLC12A9, an orphan member of the SLC12A family that has not been functionally characterized or ascribed a clear physiological role, may be involved in cardiac function. A genome-wide association study identified a locus near SLC12A9 associated with variation in the resting heart rate—a finding suggesting that genetic variation in this region may influence cardiac function and potentially contribute to cardiovascular risk [166]. This study has provided initial clues about the clinical relevance of SLC12A9, suggesting it may play an important yet unexplored role in cellular ion regulation and homeostasis.

Also, identifying specific CCCs or related molecules that could serve as biomarkers for CVD may facilitate early diagnosis, prognosis assessment, and monitoring disease progression. Studying genetic variations that affect CCC expression or function may provide insights into genetic predisposition to certain CVDs. In this respect, it is important to consider how alterations in CCC expression can contribute to the development and progression of CVD, such as heart failure, arrhythmias, atherosclerosis, and hypertension.

In conclusion, current research on SLC12A cation–chloride cotransporters in the cardiovascular system is moving beyond the kidney to explore their roles in vascular function and neuro-cardiovascular interactions. The future of SLC12A research in the cardiovascular system is multidisciplinary, moving from a focus on renal salt handling to encompass vascular biology and neuro-cardiac signaling. Understanding the interplay of these transporters across different tissues will be essential for developing effective and targeted therapies for hypertension, heart failure, and cardiovascular complications.

## Data Availability

No new data were created or analyzed in this study. Data sharing is not applicable.
